# Editorial: New Antimicrobial Peptides From Bacteria/Invertebrate Obligate Symbiotic Associations

**DOI:** 10.3389/fmicb.2022.862198

**Published:** 2022-03-08

**Authors:** András Fodor, David J. Clarke, Adler R. Dillman, Eustachio Tarasco, Selcuk Hazir

**Affiliations:** ^1^Department of Genetics, Eötvös University, Budapest, Hungary; ^2^Department of Genetics, University of Szeged, Szeged, Hungary; ^3^School of Microbiology and APC Microbiome Ireland, University College Cork, Cork, Ireland; ^4^Department of Nematology, University of California, Riverside, Riverside, CA, United States; ^5^Department of Soil, Plant and Food Sciences, University of Bari “Aldo Moro”, Bari, Italy; ^6^Institute for Sustainable Plant Protection of CNR, Bari, Italy; ^7^Department of Biology, Faculty of Arts and Sciences, Adnan Menderes University, Aydin, Turkey

**Keywords:** multidrug resistance (MDR), antimicrobial peptide (AMP), non-ribosomal templated peptides (NRP), non-ribosomal peptide synthetases (NRPS), EPN/EPB symbiosis, Legume/Rhizobium symbiosis, QSAR

Antimicrobial multidrug resistance (MDR) of the different types in prokaryotic and eukaryotic pathogenic organisms is an enormous challenge of clinical, veterinary, and plant pathogenic significance (Fodor et al., 2020). Antimicrobial peptides (AMPs) have demonstrated great potential against MDR pathogens. AMPs are polyamide molecules with an ester, thioester, or otherwise modified backbone (Ötvös and Wade, 2014). AMPs are pivotal to host defense and are widely conserved across the plant and animal kingdoms. Moreover, AMPS are often produced in prokaryote/eukaryote symbiotic associations where they bridge the innate and the adaptive immune system and provide optimal conditions for symbiosis. Efforts to maximize human benefits from AMPs antimicrobial activity include identification, quantitative structure/activity relation (QSAR) analysis of natural AMPs and derivatives (Loza et al., 2020), followed by designing, optimizing, synthesizing, and screening analogs (Fodor et al., 2020).

This Research Topic (RT) was designed as a platform for publications from separate trends in AMP research, focusing on ribosomal templated and non-ribosomal templated (NRP) AMP molecules, respectively. NRP-AMPs are synthesized via multi-enzyme thiol-template mechanisms mediated by two specific enzymes (non-ribosomal peptide synthetases (NRPS) and/or fatty acid synthase (FAS)-related polyketide synthases, PKS) (Fuchs et al., 2014; Wenski et al., 2020), encoded by biosynthetic gene clusters (BGC) (Wenski et al., 2019). In total, 10 of the 16 submitted manuscripts were accepted, of which five are reviews and five are original research papers that appeared in Frontiers Microbiology.

One review highlights new sources of AMPs and the design of peptidomimetic antimicrobial agents that can complement the defects of therapeutic peptides that have been used as a template (De Mandal et al.).

A potential source of novel NRP-AMPs is the entomopathogenic nematode/bacterium (EPN/EPB) symbiotic associations (Clarke, 2020; Tarasco and De Luca, 2021), where the prokaryotic partners (Xenorhabdus or Photorhabdus) provide optimized pathobiome conditions for the symbiosis (Ogier et al., 2020). Another of the reviews describes the history of the odilorhabdin (ODL, AMP NOSO-502), “from worms” to the current preclinical trials for the treatment of multidrug-resistant Gram-negative infections in hospitalized patients (Racine and Gualtieri). An original comparative study using the cell-free culture media of seven different EPB species indirectly proved that the NRP-AMP fabclavine (Fuchs et al., 2012, 2014; Gualtieri et al., 2012) is a presumptive nominee for curing the endodontic infections caused by MDR E. faecalis. In this study, fabclavine production was linked to a specific BGC by promoter exchange (Ozkan et al.).

In Legume/Rhizobium associations the plant directs its symbiont toward irreversible terminal differentiation, via actions of symbiosis-specific AMP-like ribosomal-templated peptides (but bacterial BacA is also required for terminal differentiation). Thus, a virulence factor of pathogenesis and effectors of innate immunity were adapted in symbiosis for the benefit of the plant partner (Kereszt et al., 2011). One review (Lima et al.) describes nodule specific cysteine-rich (NCR) legume peptides, which are exclusively produced in the symbiotic cells, and reported that those having 4–6 conserved cysteines and highly diverse amino acid sequences comprise a variety of anionic, neutral, and cationic peptides with antimicrobial activities against both bacteria and fungi. One original research article deals with two of the ~700 AMPs (NCR247 and NCR335) that exert strong antimicrobial activity on various pathogenic (including ESKAPE) bacteria. Some chimeric derivatives obtained by fusion of NCR247C with other peptide fragments proved even more efficient (Jenei et al.).

Three original research articles deal with novel AMPs obtained from Arthropoda. Mygalin (a spermidine analog) is a synthetic acylpolyamine derived from the spider Acanthoscurria gomesiana, exerting anti-Gram-negative activity with underlying mechanisms involving ROS generation and chelation of iron ions (Espinoza-Culupú et al.). Sparamosin (from mud crab Scylla paramamosain) is a peptide of 54 amino acids that contains a signal peptide. The antimicrobial activity of the synthetic mature peptide (sparamosin 26–54) exerts antimicrobial activity against a wide range of prokaryotic and eukaryotic pathogens, and anti-biofilm activity with underlying mechanisms involving ROS generation without any reported cytotoxic effects on mammalian cells (Chen et al.). Spätzle (Spz) is a dimeric ligand that responds to bacterial and fungal infections in arthropods by inducing AMP secretion. The Toll-like signaling pathway not only mediates innate immunity but modulates the homeostasis of gut microbiota (Muhammad et al.)

To date, Fusarium cannot be controlled either chemically or biologically. One original research article reported that Streptomyces huiliensis sp. nov. SCA2-4T has strong antifungal activity and genomic analysis identified 51 putative biosynthetic gene clusters of secondary metabolites. Furthermore, 10 gene clusters are involved in the biosynthesis of antimicrobial metabolites, as a biological control agent (Qi et al.). Bacteriocins are narrow spectral antimicrobial peptides that are effective against closely related competitors and have a significant drug potential. One review summarizes research efforts on biosafety of aspects of this subject (DiegoBenítez-Chao et al.).

This group of articles highlights the search for the discovery of novel AMPs that have the potential for combatting MDR pathogens *in vitro* and/or *in vivo*.

## Author Contributions

AF suggested the idea and conception of initiating that RT and then drew the conclusions allowed to write the first version of this Editorial, which, however, has not ever been materialized in the absence of the strong help of DC, who gave the most professional, formatting, and linguistic-grammar helps. DC and AD own that large scale of overlapping knowledge which made them capable of editing and handling the most MSs coming from different areas to our RT, and they provided the important add-valued to his Editorial. The special knowledge and professional skill of ET and EH were indispensably essential for the right evaluations of EPN/EPB-related papers from the aspect of the scope.

## Conflict of Interest

The authors declare that the research was conducted in the absence of any commercial or financial relationships that could be construed as a potential conflict of interest.

## Publisher's Note

All claims expressed in this article are solely those of the authors and do not necessarily represent those of their affiliated organizations, or those of the publisher, the editors and the reviewers. Any product that may be evaluated in this article, or claim that may be made by its manufacturer, is not guaranteed or endorsed by the publisher.
